# Comparing the Effect of Combining Exercise with Rosuvastatin versus Atorvastatin on Lipid Profile and Functional Capacity: A Retrospective Cohort Study

**DOI:** 10.1155/2020/7026530

**Published:** 2020-04-29

**Authors:** Sherif Eltonsy, Monique Dufour Doiron, Patrice Simard, Caroline Jose, Martin Sénéchal, Danielle R. Bouchard, Rémi LeBlanc, Mathieu Bélanger

**Affiliations:** ^1^University of Manitoba, Winnipeg, Canada; ^2^Centre de Formation Médicale du Nouveau-Brunswick, Moncton, Canada; ^3^Vitalité Health Network, Bathurst, Canada; ^4^Université de Montreal, Montreal, Canada; ^5^Maritime SPOR SUPPORT Unit, Halifax Regional Municipality, Canada; ^6^Université de Sherbrooke, Sherbrooke, Canada; ^7^University of New Brunswick, Fredericton, Canada; ^8^Université de Moncton, Moncton, Canada

## Abstract

**Background:**

Statins and exercise are recommended for managing hypercholesterolemia. However, statin types may vary in their interaction with exercise. We compared rosuvastatin versus atorvastatin combination with exercise on lipid profile and functional capacity.

**Methods:**

A retrospective cohort study using data from a 12-week cardiovascular rehabilitation program between 2014 and 2016. Statin use was determined through prescriptions, and the average exercise minutes/week were computed from exercise logs. The outcomes were changes in total cholesterol, low- and high-density lipoproteins (LDL and HDL), triglycerides, and functional capacity (6-minute walk test (6MWT)). Directed acyclic graphs were used to identify potential confounders, accounted for using multiple linear regression modeling.

**Results:**

The cohort included 282 patients from 106 atorvastatin and 176 rosuvastatin users. The average exercise minutes/week was 109.4 ± 66.1 among atorvastatin and 106.7 ± 49.1 among rosuvastatin users. Interaction models suggested that a higher number of exercise minutes/week were more favorable among atorvastatin users on total cholesterol and LDL (0.004, 95% CI: 0.001, 0.008 and 0.004, 95% CI: 0.001, 0.007, respectively) but did not reach significance for HDL and triglycerides. Rosuvastatin use was associated with greater increases in 6MWT; however, we observed no between-group differences in interaction estimates by the type of statin used.

**Conclusion:**

Rosuvastatin use could blunt the beneficial effect of exercise on LDL and total cholesterol compared to atorvastatin. No significant differences were observed in triglycerides, HDL, and functional capacity levels. Additional studies are warranted with randomized treatments and larger samples. Healthcare providers should continue prescribing statins alongside recommending exercise modalities, with a careful follow-up for rosuvastatin users.

## 1. Introduction

Hypercholesterolemia is recognized as atherogenic and can lead to numerous cardiovascular diseases (CVD) [1, 2]. Multifactorial approaches are recommended to achieve effective management of hypercholesterolemia, which includes pharmacological and lifestyle interventions [1, 3]. Among lifestyle interventions, the benefits of physical activity for hypercholesterolemia are widely recognized, including a reduction in all-cause mortality, coronary heart disease, and CVD mortality [4, 5]. The aforementioned benefits of physical activity, including physical exercise, are partially mediated by positive changes in the circulating lipoproteins, which include lowering low-density lipoprotein (LDL) and total cholesterol and increasing high-density lipoprotein (HDL) cholesterol [6, 7].

On the pharmacological intervention side, statins are the cornerstone therapy for the management of hypercholesterolemia [2, 8]. Since their introduction over thirty years ago, statins have become widely prescribed for the primary and secondary prevention of CVD, mainly for their beneficial impact on lipid metabolism and premature mortality [9, 10]. Although statins are generally well tolerated, some individuals reported a variety of skeletal muscle adverse effects ranging from muscle pain to muscle weakness [11], with markers of muscle damage such as myalgia, creatine kinase elevations, and rhabdomyolysis [12, 13]. This raises the hypothesis that use of statin exacerbates the severity of the muscle damage observed with physical activity [12, 14]. In a recent systematic review of thirteen studies, results supported the idea that intense physical activity or acute eccentric and muscle contraction might exacerbate muscle injury resulting from statin use [15]. However, this review also indicated that chronic physical activity performed at moderate intensity initiated prior to statin treatment could attenuate such side effects [15]. Further, pooled results from studies comparing the combination of physical activity and statin use versus statin monotherapy associated the combination therapy to greater improvements in exercise capacity despite showing no differences in changes in total cholesterol, LDL, HDL, and triglycerides [15].

Rosuvastatin and atorvastatin are the most commonly used types of statins, especially among older adults [16]. Statin types vary in their lipophilicity and pharmacological properties, which affect their tissue penetration, including skeletal muscle tissues [17]. Differences in the pharmacological properties of statin types could be associated to differences in their interaction with physical activity. A small trial of 28 patients with coronary artery disease performing 20 weeks of aerobic exercise observed a greater increase in HDL levels among patients randomized to rosuvastatin combined with exercise than patients randomized to atorvastatin combined with exercise [18]. Given the small sample size of this study and the use of a very low dose of rosuvastatin (mean dose = 2.9 mg in contrast with the typical dosage range of 5 to 40 mg/day) [18], it is possible that we can expect even larger differences of how exercise interacts with different types of statins in practice.

We hypothesized that the interaction between exercise and statins is partially dependent on the properties of the statin used by patients, where the higher efficacy statin (i.e., rosuvastatin) would be more likely to be associated with additional benefits when combined with exercise. Therefore, in the current study, we present a real-world comparison of rosuvastatin versus atorvastatin combination with exercise to examine the impact of the interaction between the type of statin used and exercise. The main outcomes studied were lipid profile and functional capacity.

## 2. Materials and Methods

### 2.1. Study Design and Source of Data

The methods used in the study were described in details in Eltonsy et al. [19]. A population-based retrospective cohort study was conducted using data from the electronic records of participants in the Cardiac Wellness Program, a cardiac rehabilitation program established in Moncton, New Brunswick, Canada. The Cardiac Wellness Program provides services to cardiac patients and patients at risk of CVD in the greater Moncton area. The Cardiac Wellness Program is affiliated with the Canadian Association of Cardiovascular Prevention and Rehabilitation (CACPR) [20]. Once a patient is admitted into the program, an electronic record is created based on the patient's hospital services information. Additional information is obtained through individual interviews with the program's staff, laboratory tests results, and program utilization (e.g., details of physical exercise performed) over the 12 weeks of cardiovascular rehabilitation. This study was approved by the Research Ethics Committee of the Vitalité Health Network.

### 2.2. Participant Selection

The cohort inclusion criteria for the main analyses were minimal age of 35 years at admission, a recorded admission date between January 2014 and June 2016, and completing the discharge reassessment at the end of program. We excluded patients with missing data on any of the primary exposures of interest (i.e., medications used and exercise performed during the program), as well as patients with missing data on all of the study outcomes (functional capacity and lipid profile). A total of 807 patients were first examined to be considered in the study analyses (Supplementary 1). From these, we excluded 404 patients that had missing data on all primary outcomes, the medications used, or the exercise minutes performed during the program. We excluded 104 nonusers of statins, as well as 10 simvastatin and 7 pravastatin users. The final sample analyzed for the primary cohort included 282 patients for whom we had the necessary information on their exposures and at least one of the primary outcomes, categorized into 176 rosuvastatin users and 106 atorvastatin users.

### 2.3. Exposure Assessment

The 12-week cardiac rehabilitation program was based on recommendations of the CACPR [21]. It included an individualized exercise plan based on patients' conditions and needs. Individual sessions generally consisted of a brief warm-up, 30-45 minutes of exercise using a variety of aerobic modalities, including treadmills, stationary cycles, arm ergocycles, elliptical trainers, and rowers, and followed by a brief cooldown and stretching. Exercise intensity was prescribed following the Karvonen method, with a heart rate typically ranging between 45 and 85% of heart rate reserve, based on the referral diagnosis and patient's exercise capacity [22]. We quantified the amount of exercise as the average number of minutes of exercise performed per week during the 12-week cardiac rehabilitation program. Exposure to rosuvastatin and atorvastatin (in brand or generic form) was determined through a search among all recorded medications used at admission to the Cardiac Wellness Program.

### 2.4. Outcomes Definition

The study outcomes were the changes, from admission to discharge, in lipid profile, including total cholesterol (mmol/L), triglycerides (TG) (mmol/L), low-density lipoprotein (LDL) (mmol/L), and high-density lipoprotein (HDL) (mmol/L), and functional capacity. Lipid profile was measured at the hospital laboratory in fasting state. Functional capacity was measured as the distance walked over a total of six minutes using the standard 6-minute walk test (6MWT) [23]. The 6MWT was carried out on a 30-meter hallway. One well-trained kinesiologist/exercise training professional supervised the test. Patients were instructed to walk the length of the hallway as many times as possible in the allotted period of six minutes. The patients were allowed to stop and rest during the test but were instructed to resume walking as soon as they felt able to do so.

### 2.5. Confounding Variables

Directed acyclic graphs (DAG) were used to identify potential confounders and specify variables to be included in the models to minimize bias [24]. Three classes of potential confounders were included in the analysis: first, sociodemographic and clinical variables measured at admission, including age (years), sex, tobacco smoking (non-, previous, or current smoker), weight at admission (kg), and systolic and diastolic blood pressure (mmHg); second, medications used at admission, including other cholesterol-lowering medications (yes/no), anticoagulants (yes/no), antiplatelets (yes/no), cardiovascular medications (including antihypertensive and angina medications) (yes/no), oral antidiabetics (yes/no), and insulin (yes/no); and third, exercise-related variables, including adherence to exercise schedule (percentage of weekly sessions performed/prescribed) and 6MWT measured at admission (meters).

### 2.6. Statistical Analysis

Descriptive statistics for the characteristics of patients were calculated and compared between groups of rosuvastatin and atorvastatin users. The outcomes were first analyzed in crude models using multiple linear regression models, with exposures and other variables separately included in the models. Afterwards, the outcomes were analyzed in simple interaction models, with statin type use (atorvastatin as reference), average exercise minutes/week, and their interaction product terms serving as independent variables [25]. Subsequently, fully adjusted models were developed by adding potentially confounding variables. Figure plots representing adjusted change in outcomes as a function of average exercise minutes/week and statin type use were depicted. Given the proportion of missing data, we performed sensitivity analyses accounting for missing data. We used the full information maximum-likelihood method for missing data, generating crude and fully adjusted multiple linear regression models, in a similar process as the primary analyses described above [26].

In the pre hoc sample size calculation using a type I error of 0.05 and 80% power, a total sample size of 280 patients (in 1 : 1 groups) was estimated to be sufficient to detect a 0.2 mmol/L change in LDL, and 300 patients was estimated to be sufficient to detect a 10% increase in functional capacity (6MWT). The actual study power was lower than this due to unbalanced sampling caused by the fewer number of atorvastatin users versus rosuvastatin users. Statistical analyses were conducted using the SAS software, version 9.4 (SAS Institute Inc., Cary, NC), and Dagitty for causal diagrams [27].

## 3. Results

Among rosuvastatin users, 72 (40.9%) were low- to moderate-intensity users (5-10 mg) and 104 (59.1%) were high-intensity users (20-40 mg) (Table 1). Among atorvastatin users, 33 (31.1%) were low- to moderate-intensity users (10-20 mg) and 73 (68.9%) were high-intensity users (40-80 mg).

The overall mean age, distribution of sex, smoking history, baseline blood pressure, and SF-36 scores were similar in both groups (Table 1). At admission, levels of HbA_1c_ were similar between rosuvastatin and atorvastatin users. Levels of triglycerides and total cholesterol were similar; however, rosuvastatin users had significantly lower LDL and higher HDL levels compared to atorvastatin. Rosuvastatin users were also slightly heavier than atorvastatin users. The use of other medications was comparable between atorvastatin and rosuvastatin users. The use of other cholesterol-lowering medications was low, but their use was more common among rosuvastatin users. The use of oral antidiabetics and insulin was higher among rosuvastatin users versus atorvastatin users.

At admission, both groups performed similarly on their 6MWT. Atorvastatin users performed few more minutes of exercise per week during the 12-week rehabilitation program than rosuvastatin users. However, rosuvastatin users were more adherent to their prescribed exercise program compared to atorvastatin users (80.8% vs. 73.7% exercise sessions attended, respectively).

### 3.1. Primary Outcomes

**Figure 1:**
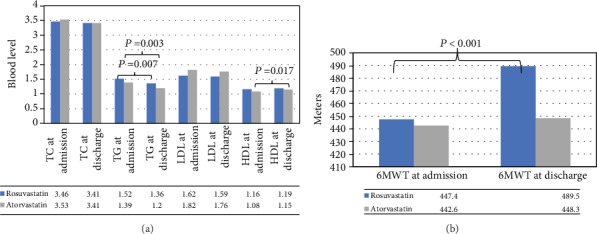
Calculated (a) lipid profile levels and (b) 6MWT at admission and discharge for rosuvastatin and atorvastatin users.

From admission in the 12-week rehabilitation program to discharge, no clinically meaningful changes in lipid profile parameters were observed among rosuvastatin users (total cholesterol change from 3.46 mmol/L to 3.41 mmol/mol, *P* value = 0.805, LDL change from 1.62 mmol/L to 1.59 mmol/mol, *P* value = 0.549, and HDL change from 1.16 mmol/L to 1.19 mmol/mol, *P* value = 0.141) except for triglycerides (change from 1.52 mmol/L to 1.36 mmol/mol, *P* value = 0.007) (Figure 1(a)). Among atorvastatin users, significant changes were observed in both HDL and triglycerides (HDL change from 1.08 mmol/L to 1.15 mmol/mol, *P* value = 0.017, and triglycerides change from 1.39 mmol/L to 1.20 mmol/mol, *P* value = 0.003) but not for other parameters (total cholesterol change from 3.53 mmol/L to 3.41 mmol/mol, *P* value = 0.682, LDL change from 1.82 mmol/L to 1.76 mmol/mol, *P* value = 0.782) (Figure 1(a)). Only the group of patients using rosuvastatin had a significant improvement in 6MWT distance (increase of 5.7 meters among atorvastatin users (*P* value = 0.484) versus 42.1 meters among rosuvastatin users (*P* value < 0.001)) (Figure 1(b)).

In the crude models for lipid profile parameters, more minutes of exercise per week were related to increases in HDL. Neither type of statin used nor exercise minutes/week reached significance with the other lipid parameters measured (Tables 2–5). In the simple interaction models for changes in lipid profile parameters, we did not detect differences in the association between the average exercise minutes per week and changes in lipid parameters as a function of type of statin used (i.e., interaction between type of statin used and average exercise minutes/week). However, after adjusting for potential confounders (fully adjusted models, Tables 2 and 3), interaction terms suggested that a higher number of exercise minutes per week were more favorable among the group of atorvastatin users for the outcomes of total cholesterol and LDL (Tables 2 and 3 and Supplementary 2). For the outcome of functional capacity (i.e., change in 6MWT), rosuvastatin use was associated with greater increases in 6MWT in all models. However, the estimated effect of average exercise minutes/week was not different between the types of statin used by patients (Table 6 and Supplementary 3).

In sensitivity analyses accounting for missing data, interaction between the type of statin used and average exercise minutes/week was significant for the outcome of LDL, suggesting a more favorable effect of exercise among atorvastatin users than rosuvastatin users (*β* 0.002, 95% CI 0.001, 0.004), but not for total cholesterol (*β* 0.001, 95% CI -0.001, 0.003). These results are similar in part to the results from primary analyses, and all results from sensitivity analyses for the other study outcomes were similar to observations from the primary analyses (data not shown, available upon request).

## 4. Discussion

In the current study of patients enrolled in a 12-week cardiac rehabilitation program, we observed a difference in the effect of exercise performed over the 12-week period between patients using rosuvastatin and those using atorvastatin. Specifically, although we observed that using rosuvastatin vs. atorvastatin was associated with greater improvements in total cholesterol and LDL levels, we also noted that using rosuvastatin was associated with an attenuation of the effect of exercise on these outcomes. Those results suggest that the difference in effectiveness attributed to different types of statins, whereby rosuvastatin appears more effective in lowering LDL cholesterol [28], may be attenuated by engaging in more exercise. In the current study, using rosuvastatin was associated with a blunting effect on the expected benefits from exercise on total cholesterol and LDL level reductions. Those observations could have direct clinical significance as patients at risk of CVD are commonly prescribed rosuvastatin to control their lipid levels and at the same time advised to exercise, both of which are independently proven to lower CVD risk. The current study did not identify other parameters of lipid profile (i.e., HDL and triglycerides) or functional capacity that suggested further interaction between the effect of exercise and the type of statin used by patients.

To the best of our knowledge, the current study represents one of the largest studies to date on the topic. Our results are partially concordant with the results from Toyama et al. [18] who also did not observe a significant difference in changes in triglycerides and functional capacity when comparing atorvastatin and rosuvastatin. However, their study reported a significant change in HDL (*P* < 0.05) in favour of rosuvastatin use and nonsignificant change in LDL levels. The discrepancies in results from Toyama et al. [18] and the current study may be attributable to differences in the studies designs and inclusion criteria. For example, restriction to only 28 patients with coronary artery disease versus 282 CVD patients and patients at risk in the current study, exercise training was supervised once weekly in Toyama et al.'s study [18], whereas patients received continuous supervision of their exercise training in the current study. Moreover, the previous study included new users of statins at low doses, whereas the current study included new and current users of statins, with an average daily dose of atorvastatin and rosuvastatin in concordance with real-world common prescribing practice.

The findings of the current study necessitate exploring their potential underlying mechanisms. The methyl sulfonamide moiety of rosuvastatin adds a relative hydrophilicity to the molecule compared to atorvastatin. Thus, rosuvastatin is relatively hydrophilic, compared with a lipophilic atorvastatin [29]. The importance of this characteristic resides in the fact that more lipophilic molecules can enter different cell membranes by passive diffusion, having unrestricted access to different cell types, including skeletal muscle tissues. On the contrary, statins with greater hydrophilicity would have lower rates of passive diffusion. Studies have demonstrated an uptake of rosuvastatin into hepatocytes via both passive diffusion and active transport, but predominantly the latter [29, 30]. However, we did not observe a significant difference between rosuvastatin and atorvastatin on physical function, which can potentially be attributed to the two molecules being close on the relative hydrophilicity scale since rosuvastatin is the least hydrophilic and atorvastatin is the least hydrophobic among all statins [30].

The mechanism through which rosuvastatin may blunt exercise benefits on LDL compared to atorvastatin requires additional investigation. A prolonged elimination half-life is considered an advantage for a statin, allowing for a lengthier inhibition of liver enzyme through the dose interval and maximum upregulation of hepatic LDL receptors [17, 29]. At approximately 20 hours, rosuvastatin has the longest elimination half-life, compared to 14 hours for atorvastatin [29]. Theoretically, if statins blunt exercise benefits on LDL through a competing mechanism, a differential effect in favour of the shorter half-life atorvastatin may be more likely, as observed in the current study.

### 4.1. Strengths and Limitations

Strengths of the current study include examining statin-exercise interaction among individuals enrolled in a cardiac rehabilitation program, a sample representative of real-world patients attending cardiac rehabilitation programs. While participants in this cardiac rehabilitation program are not representative of the general population, their health condition reflects that of the population typically targeted by statins therapy, who would mostly benefit from both statins and exercise. Through comparing two medications that have similar indication, we were able to minimize the potential bias introduced by the confounding by indication. Moreover, we adjusted for several potentially confounding variables, including smoking and different medications classes used for chronic diseases. Finally, the sample size in the current study is relatively large in comparison to similar published studies on this specific topic.

The results should nevertheless be interpreted with consideration of the following limitations. We did not have data on the acute effects of statin use. Due to the nonrandomized nature of the study, we were unable to reduce bias that could be caused by unmeasured factors. However, data recording was not influenced by the study objectives; therefore, measurement errors in the recording of data are likely to be similar for rosuvastatin and atorvastatin users such that if bias occurred, it most likely was nondifferential. We did not have data of the severity of the underlying cardiac disease. We did not have data on the duration of statin use prior to enrollment. We expect that CVD patients would have started statin use before enrollment in the program, which can be relatively observed in their controlled lipid profile levels at admission. However, rosuvastatin and atorvastatin users shared similar disease profiles at admission; therefore, an additional benefit from exercise—if independent of the statin type used—should have been observed in a balanced manner. Data on dietary changes, exercise intensity, and other physical activities performed outside of the rehabilitation program was not available. Statin use was measured using the recorded prescriptions data and patient interviews, with actual intake of the medication being unknown. However, discharge interviews showed similar use for statins among participants as at admission. The objectives of the current study did not comprise assessment of exercise and statins interaction as a class of medication in itself or the effect of statins doses on musculoskeletal injuries, but rather to compare two widely used statin types in combination with exercise modalities. It is possible that both types of statins interact with exercise [10, 15], but a group of nonusers of statins were not included in the study. Although we achieved balanced groups by comparing two treatments with similar indication, the potential for residual confounding as a result of indication bias cannot be ruled out. We did not have enough power to analyze different doses of statin types, but the difference in doses was not significant between groups. Finally, the data were retrieved from one center and only 50% of patients had full data to be included in the final analysis, affecting the generalizability of our results.

## 5. Conclusions

In conclusion, evidence from the current study on the prolonged (12 weeks or more) exposure to a combination of statins and exercise among cardiac rehabilitation population suggest that rosuvastatin use could possibly blunt the beneficial effect of exercise on LDL and total cholesterol compared to atorvastatin use. No significant differences were observed in the triglycerides, HDL, and functional capacity levels. Given the importance of optimizing the treatment strategies for CVD patients and patients at risk, additional studies are warranted in this area with randomized treatments and large sample sizes. We encourage healthcare practitioners to continue prescribing statins concomitantly alongside recommending physical exercise modalities, with a careful follow-up for the lipid profile of rosuvastatin users.

## Figures and Tables

**Table 1:** Characteristics of patients included in the analyses according to statin type used.

Sociodemographic and clinical variables measured at admission	Atorvastatin users (106 patients)	Rosuvastatin users (176 patients)	*P* value
	No. (%) or mean ± SD	No. (%) or mean ± SD	
Age (years)	65.9 ± 10.4	65.8 ± 9.6	0.94
Sex (male)	75 (70.8)	120 (68.2)	0.68
Tobacco smoking			
Nonsmoker	54 (51.4)	83 (47.7)	0.54
Previous smoker	42 (40.0)	78 (44.8)	0.43
Current smoker	9 (8.6)	13 (7.5)	0.74
Weight (kg)	87.3 ± 19.2	90.9 ± 21.3	0.16
Blood pressure (mmHg)			
Systolic BP	118.3 ± 15.7	120.8 ± 14.5	0.18
Diastolic BP	68.6 ± 9.8	68.6 ± 9.4	1.0
Lipids (mmol/L)			
Total cholesterol	3.53 ± 0.73	3.46 ± 0.64	0.39
Triglycerides	1.39 ± 0.58	1.52 ± 0.78	0.14
LDL	1.82 ± 0.58	1.62 ± 0.50	0.002
HDL	1.08 ± 0.27	1.16 ± 0.30	0.03
HbA_1c_ (mmol/mol)	43 ± 9.9	43 ± 9.3	0.95
HbA_1c_ (%)	6.10 ± 0.91	6.09 ± 0.85	0.95
6-minute walk test (meters)	442.6 ± 136.3	447.4 ± 113.2	0.75
SF-36 score	73.8 ± 25.7	76.3 ± 24.5	0.42
Medications used			
Cardiovascular medications	102 (96.2)	163 (92.6)	0.22
Anticoagulants	18 (17.0)	24 (13.6)	0.44
Antiplatelets	91 (85.9)	148 (84.1)	0.68
Other cholesterol-lowering medications	7 (6.6)	18 (10.2)	0.30
Oral antidiabetics	22 (20.8)	47 (26.7)	0.26
Insulin	7 (6.6)	16 (9.1)	0.46
Statin doses^∗^			
Low to moderate intensity	33 (31.1)	72 (40.9)	0.09
High intensity	73 (68.9)	104 (59.1)	0.09
Exercise performed during the 12-week cardiac rehabilitation program			
Total minutes of exercise	1042.4 ± 827.3	1088.7 ± 665.8	0.61
Adherence (%) to exercise schedule (weekly sessions performed/prescribed)	73.7 ± 30.4	80.8 ± 28.7	0.05
Average of weekly exercise minutes	109.4 ± 66.1	106.7 ± 49.1	0.69

HbA_1c_: glycated haemoglobin; SF-36: 36-Item Short-Form Health Survey; LDL: low-density lipoprotein; HDL: high-density lipoprotein; BP: blood pressure. ^∗^Stone et al. [8].

**Table 2:** Crude and adjusted linear regression coefficients and 95% confidence intervals for changes in total cholesterol.

Variable	Change in total cholesterol					
	Crude model		Simple interaction model		Fully adjusted model	
	*β*	95% CI	*β*	95% CI	*β*	95% CI
Intercept	NA	NA	0.059	(-0.209, 0.328)	0.660	(-1.497, 2.818)
Atorvastatin use (yes)	Reference	Reference	Reference	Reference	Reference	Reference
Rosuvastatin use (yes)	0.026	(-0.134, -0.186)	-0.133	(-0.505, 0.239)	-0.612	(-1.177, -0.478)
Average exercise minutes/week (minutes)	-0.0001	(-0.001, 0.001)	-0.001	(-0.003, 0.001)	-0.002	(-0.005, 0.001)
Rosuvastatin use^∗^ Average exercise interaction	NA	NA	0.001	(-0.001, 0.004)	0.004	(0.001, 0.008)
Age (years)	0.002	(-0.007, 0.011)	NA	NA	-0.002	(-0.018, 0.014)
Sex (male)	0.084	(-0.078, 0.247)	NA	NA	0.267	(-0.063, 0.596)
Tobacco smoking (previous/current smoker)	-0.005	(-0.135, 0.125)	NA	NA	-0.089	(-0.321, 0.142)
Other cholesterol-lowering medications (yes)	0.117	(-0.160, 0.394)	NA	NA	0.147	(-0.300, 0.595)
Anticoagulants (yes)	-0.012	(-0.242, 0.217)	NA	NA	-0.220	(-0.730, 0.289)
Antiplatelets (yes)	0.158	(-0.066, 0.382)	NA	NA	-0.143	(-0.596, 0.310)
Cardiovascular medications (yes)	0.159	(-0.169, 0.488)	NA	NA	0.213	(-0.229, 0.656)
Oral antidiabetics (yes)	-0.004	(-0.188, 0.181)	NA	NA	0.094	(-0.196, 0.383)
Insulin (yes)	-0.125	(-0.454, 0.203)	NA	NA	-0.455	(-0.956, 0.047)
Adherence (%) to exercise schedule (weekly sessions performed/prescribed)	-0.001	(-0.005, 0.004)	NA	NA	0.004	(-0.005, 0.012)
Weight (kg)	0.001	(-0.004, 0.004)	NA	NA	-0.004	(-0.110, 0.004)
Systolic BP (mmHg)	0.002	(-0.002, 0.007)	NA	NA	-0.002	(-0.012, 0.009)
Diastolic BP (mmHg)	0.004	(-0.004, 0.013)	NA	NA	0.006	(-0.009, 0.022)
6MWT at admission (meters)	-0.001	(-0.002, 0.0003)	NA	NA	-0.001	(-0.003, 0.000)

**Table 3:** Crude and adjusted linear regression coefficients and 95% confidence intervals for changes in LDL.

Variable	Change in LDL					
	Crude model		Simple interaction model		Fully adjusted model	
	*β*	95% CI	*β*	95% CI	*β*	95% CI
Intercept	NA	NA	0.159	(-0.041, 0.349)	0.804	(-0.623, 2.232)
Atorvastatin use (yes)	Reference	Reference	Reference	Reference	Reference	Reference
Rosuvastatin use (yes)	0.002	(-0.113, 0.118)	-0.179	(-0.449, 0.089)	-0.518	(-0.902, -0.134)
Average exercise minutes/week (minutes)	-0.0004	(-0.001, 0.001)	-0.001	(-0.002, 0.001)	-0.002	(-0.004, -0.001)
Rosuvastatin use^∗^ Average exercise interaction	NA	NA	0.001	(-0.001, 0.003)	0.004	(0.001, 0.007)
Age (years)	0.0001	(-0.006, 0.006)	NA	NA	0.005	(-0.006, 0.015)
Sex (male)	0.074	(-0.043, 0.191)	NA	NA	0.275	(0.057, 0.493)
Tobacco smoking (previous/current smoker)	0.004	(-0.094, 0.101)	NA	NA	0.003	(-0.161, 0.167)
Other cholesterol-lowering medications (yes)	0.033	(-0.167, 0.233)	NA	NA	0.099	(-0.197, 0.396)
Anticoagulants (yes)	0.031	(-0.134, 0.197)	NA	NA	-0.205	(-0.548, 0.138)
Antiplatelets (yes)	0.099	(-0.065, 0.264)	NA	NA	-0.124	(-0.430, 0.182)
Cardiovascular medications (yes)	-0.033	(-0.269, 0.204)	NA	NA	-0.079	(-0.371, 0.214)
Oral antidiabetics (yes)	0.005	(-0.128, 0.138)	NA	NA	0.049	(-0.144, 0.241)
Insulin (yes)	-0.025	(-0.275, 0.226)	NA	NA	-0.245	(-0.599, 0.109)
Adherence (%) to exercise schedule (weekly sessions performed/prescribed)	-0.002	(-0.005, 0.002)	NA	NA	0.001	(-0.005, 0.005)
Weight (kg)	0.001	(-0.002, 0.004)	NA	NA	-0.002	(-0.007, 0.003)
Systolic BP (mmHg)	0.001	(-0.002, 0.005)	NA	NA	-0.005	(-0.012, 0.002)
Diastolic BP (mmHg)	0.004	(-0.001, 0.010)	NA	NA	0.008	(-0.003, 0.018)
6MWT at admission (meters)	-0.001	(-0.001, 0.0001)	NA	NA	-0.001	(-0.002, -0.0001)

6MWT: 6-minute walk test; LDL: low-density lipoprotein; HDL: high-density lipoprotein; BP: blood pressure.

**Table 4:** Crude and adjusted linear regression coefficients and 95% confidence intervals for changes in HDL.

Variable	Change in HDL					
	Crude model		Simple interaction model		Fully adjusted model	
	*β*	95% CI	*β*	95% CI	*β*	95% CI
Intercept	NA	NA	-0.029	(-0.124, 0.066)	0.326	(-0.354, 1.007)
Atorvastatin use (yes)	Reference	Reference	Reference	Reference	Reference	Reference
Rosuvastatin use (yes)	-0.036	(-0.093, 0.022)	0.013	(-0.119, 0.145)	0.016	(-0.162, 0.194)
Average exercise minutes/week (minutes)	0.001	(0.0005, 0.001)	0.001	(0.0001, 0.001)	0.001	(0.0001, 0.002)
Rosuvastatin use^∗^ Average exercise interaction	NA	NA	-0.0004	(-0.001, 0.001)	-0.0004	(-0.002, 0.001)
Age (years)	0.001	(-0.002, 0.004)	NA	NA	0.001	(-0.004, 0.006)
Sex (male)	0.029	(-0.294, 0.088)	NA	NA	0.058	(-0.046, 0.162)
Tobacco smoking (previous/current smoker)	0.035	(-0.011, 0.082)	NA	NA	0.034	(-0.039, 0.107)
Other cholesterol-lowering medications (yes)	0.012	(-0.089, 0.112)	NA	NA	0.045	(-0.096, 0.186)
Anticoagulants (yes)	-0.084	(-0.164, -0.004)	NA	NA	-0.083	(-0.244, 0.078)
Antiplatelets (yes)	0.037	(-0.044, 0.119)	NA	NA	0.002	(-0.141, 0.145)
Cardiovascular medications (yes)	0.115	(-0.002, 0.233)	NA	NA	0.051	(-0.089, 0.190)
Oral antidiabetics (yes)	-0.029	(-0.095, 0.038)	NA	NA	0.015	(-0.076, 0.106)
Insulin (yes)	-0.070	(-0.189, 0.048)	NA	NA	-0.102	(-0.260, 0.056)
Adherence (%) to exercise schedule (weekly sessions performed/prescribed)	0.0001	(-0.002, 0.002)	NA	NA	-0.001	(-0.004, 0.001)
Weight (kg)	-0.0001	(-0.002, 0.001)	NA	NA	-0.0001	(-0.002, 0.002)
Systolic BP (mmHg)	-0.001	(-0.002, 0.001)	NA	NA	0.0001	(-0.003, 0.003)
Diastolic BP (mmHg)	-0.004	(-0.006, -0.001)	NA	NA	-0.004	(-0.009, 0.001)
6MWT at admission (meters)	-0.0001	(-0.0003, 0.0003)	NA	NA	-0.0003	(-0.0008, 0.0001)

**Table 5:** Crude and adjusted linear regression coefficients and 95% confidence intervals for changes in triglycerides.

Variable	Change in triglycerides					
	Crude model		Simple interaction model		Fully adjusted model	
	*β*	95% CI	*β*	95% CI	*β*	95% CI
Intercept	NA	NA	-0.179	(-0.385, 0.026)	-0.429	(-1.620, 0.763)
Atorvastatin use (yes)	Reference	Reference	Reference	Reference	Reference	Reference
Rosuvastatin use (yes)	0.037	(-0.085, 0.158)	0.154	(-0.130, 0.438)	-0.164	(-0.475, 0.148)
Average exercise minutes/week (minutes)	-0.0002	(-0.001, 0.001)	0.0003	(-0.001, 0.002)	0.0001	(-0.002, 0.001)
Rosuvastatin use^∗^ Average exercise interaction	NA	NA	-0.001	(-0.003, 0.001)	0.001	(-0.001, 0.003)
Age (years)	0.002	(-0.005, 0.008)	NA	NA	-0.005	(-0.014, 0.004)
Sex (male)	0.019	(-0.106, 0.144)	NA	NA	-0.051	(-0.233, 0.131)
Tobacco smoking (previous/current smoker)	0.077	(-0.175, 0.022)	NA	NA	-0.163	(-0.291, -0.035)
Other cholesterol-lowering medications (yes)	0.110	(-0.102, 0.321)	NA	NA	0.105	(-0.142, 0.353)
Anticoagulants (yes)	0.077	(-0.094, 0.248)	NA	NA	0.304	(0.022, 0.585)
Antiplatelets (yes)	0.059	(-0.113, 0.230)	NA	NA	0.099	(-0.150, 0.350)
Cardiovascular medications (yes)	-0.150	(-0.400, 0.100)	NA	NA	0.012	(-0.232, 0.257)
Oral antidiabetics (yes)	-0.019	(-0.160, 0.122)	NA	NA	-0.091	(-0.251, 0.069)
Insulin (yes)	-0.040	(-0.292, 0.211)	NA	NA	0.013	(-0.264, 0.290)
Adherence (%) to exercise schedule (weekly sessions performed/prescribed)	-0.001	(-0.005, 0.002)	NA	NA	-0.002	(-0.006, 0.003)
Weight (kg)	-0.0001	(-0.003, 0.003)	NA	NA	0.0002	(-0.004, 0.004)
Systolic BP (mmHg)	0.003	(-0.001, 0.006)	NA	NA	0.007	(0.001, 0.012)
Diastolic BP (mmHg)	0.006	(-0.0001, 0.012)	NA	NA	0.0004	(-0.008, 0.009)
6MWT at admission (meters)	0.0001	(-0.001, 0.001)	NA	NA	0.0002	(-0.001, 0.001)

6MWT: 6-minute walk test; LDL: low-density lipoprotein; HDL: high-density lipoprotein; BP: blood pressure.

**Table 6:** Crude and adjusted linear regression coefficients and 95% confidence intervals for changes in 6MWT.

Variable	Change in 6MWT					
	Crude model		Simple interaction model		Fully adjusted model	
	*β*	95% CI	*β*	95% CI	*β*	95% CI
Intercept	NA	NA	5.131	(-24.940, 35.202)	114.935	(-19.333, 249.202)
Atorvastatin use (yes)	Reference	Reference	Reference	Reference	Reference	Reference
Rosuvastatin use (yes)	36.440	(18.090, 54.791)	47.328	(5.589, 89.067)	46.021	(2.866, 89.176)
Average exercise minutes/week (minutes)	-0.062	(-0.225, 0.102)	0.004	(-0.204, 0.212)	-0.082	(-0.297, 0.134)
Rosuvastatin use^∗^ Average exercise interaction	NA	NA	-0.094	(-0.408, 0.219)	-0.043	(-0.363, 0.277)
Age (years)	-0.298	(-1.243, 0.647)	NA	NA	-0.009	(-1.067, 1.049)
Sex (male)	9.749	(-9.798, 29.295)	NA	NA	14.808	(-7.148, 36.763)
Tobacco smoking (previous/current smoker)	-1.037	(-16.426, 14.352)	NA	NA	1.571	(-14.644, 17.787)
Other cholesterol-lowering medications (yes)	5.982	(-25.776, 37.741)	NA	NA	9.326	(-21.640, 40.293)
Anticoagulants (yes)	-3.371	(-32.859, 26.118)	NA	NA	-7.992	(-40.845, 24.860)
Antiplatelets (yes)	12.388	(-19.310, 44.086)	NA	NA	16.740	(-19.159, 52.640)
Cardiovascular medications (yes)	-14.115	(-50.799, 22.569)	NA	NA	-9.581	(-47.003, 27.842)
Oral antidiabetics (yes)	-31.361	(-51.964, -10.757)	NA	NA	-25.519	(-47.835, -3.203)
Insulin (yes)	-19.058	(-53.710, 15.593)	NA	NA	-10.874	(-45.502, 23.753)
Adherence (%) to exercise schedule (weekly sessions performed/prescribed)	-0.169	(-0.682, 0.345)	NA	NA	-0.329	(-0.865, 0.206)
Weight (kg)	-0.298	(-0.769, 0.172)	NA	NA	-0.361	(-0.906, 0.185)
Systolic BP (mmHg)	-0.128	(-0.718, 0.462)	NA	NA	-0.512	(-1.239, 0.214)
Diastolic BP (mmHg)	0.240	(-0.736, 1.216)	NA	NA	0.143	(-0.986, 1.272)

6MWT: 6-minute walk test; BP: blood pressure.

## Data Availability

The datasets used and/or analysed during the current study are available from the corresponding author on reasonable request.

## Supplementary Materials

Supplementary 1 Flowchart for patients included in the study.

Supplementary 2 Adjusted model estimates and 95% CI of change in (A) total cholesterol, (B) LDL, (C) HDL, and (D) triglycerides (in mmol/L) from baseline to the end of 12-week cardiac rehabilitation program by average exercise minutes per week and statin type used (using parametric test).

Supplementary 3 Adjusted model estimates and 95% CI of change in 6-minute walk test (6MWT) from baseline to end of 12-week cardiac rehabilitation program by average exercise minutes per week and statin type used (using parametric test).
