# Supplementary material for: Comparing the Effect of Combining Exercise with Rosuvastatin versus Atorvastatin on Lipid Profile and Functional Capacity: A Retrospective Cohort Study
Source: Biomed Res Int. 2020 Apr 29;2020:7026530. doi: 10.1155/2020/7026530 (PMC7210511; doi:10.1155/2020/7026530)
Supplement: Supplementary 1 — Flowchart for patients included in the study. [file 7026530.f1.docx]

**176 Rosuvastatin users**

**10** Simvastatin and **7** Pravastatin users

**Figure S1.** Flowchart for patients included in the study

**<**

**807** patients admitted to the cardiac rehabilitation program between January 2014 and June 2016

**479** patients

**466** patients

**403** patients

**13** admissions with missing data on medications used

**63** admissions with missing data on physical exercise performed during the program

**328** patients with missing data on primary outcomes

**282** patients

**106 Atorvastatin users**

**104** Statins non-users

**299** patients
