# Supplementary material for: Comparing the Effect of Combining Exercise with Rosuvastatin versus Atorvastatin on Lipid Profile and Functional Capacity: A Retrospective Cohort Study
Source: Biomed Res Int. 2020 Apr 29;2020:7026530. doi: 10.1155/2020/7026530 (PMC7210511; doi:10.1155/2020/7026530)
Supplement: Supplementary 2 — Adjusted model estimates and 95% CI of change in (A) total cholesterol, (B) LDL, (C) HDL, and (D) triglycerides (in mmol/L) from baseline to the end of 12-week cardiac rehabilitation program by average exercise minutes per week and statin type used (using parametric test). [file 7026530.f2.docx]

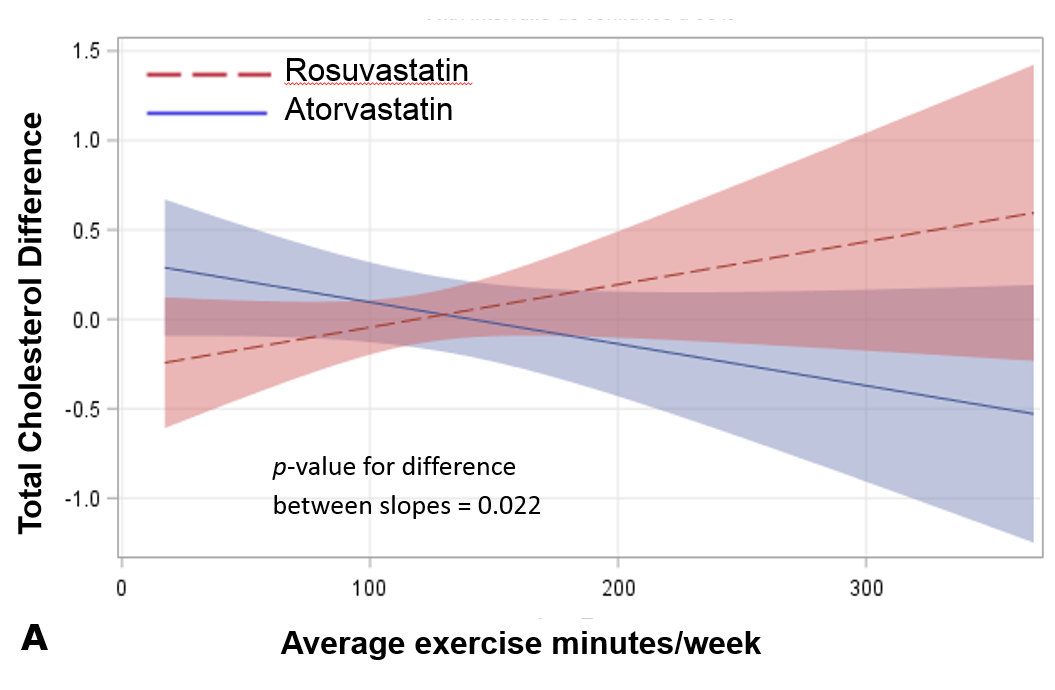

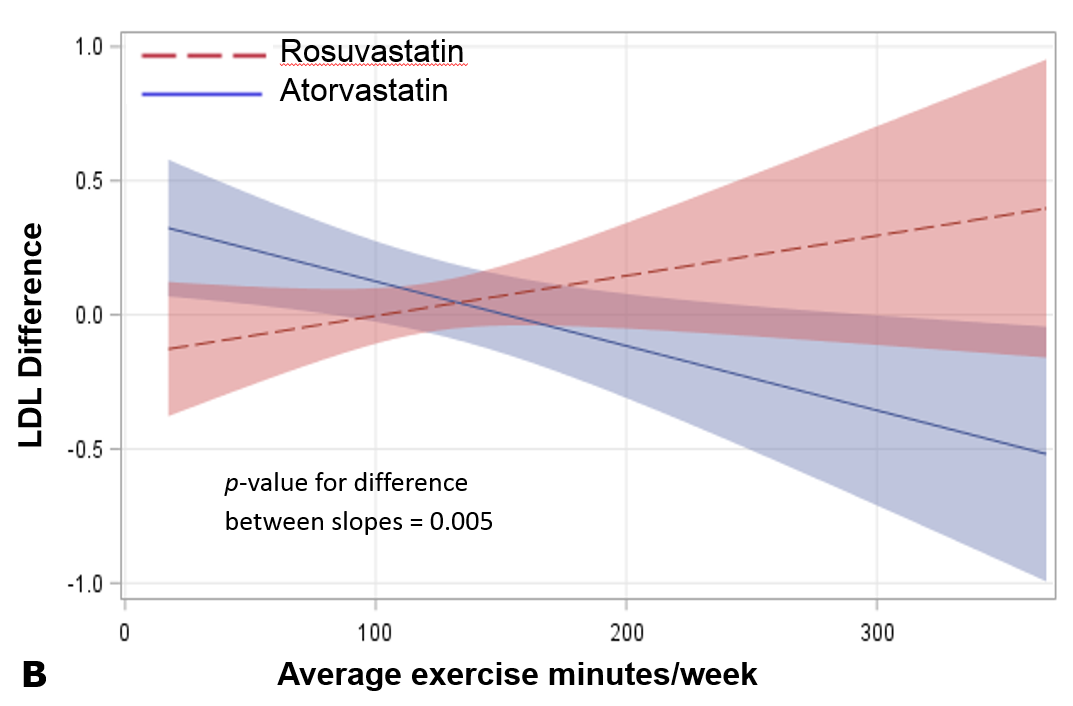


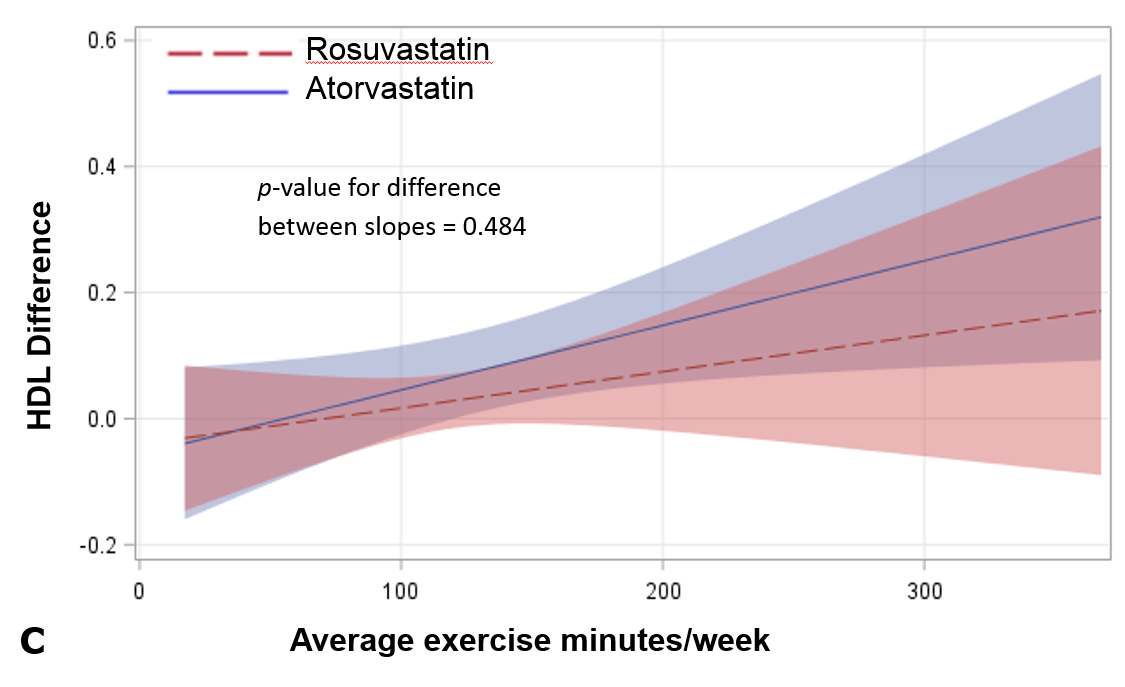

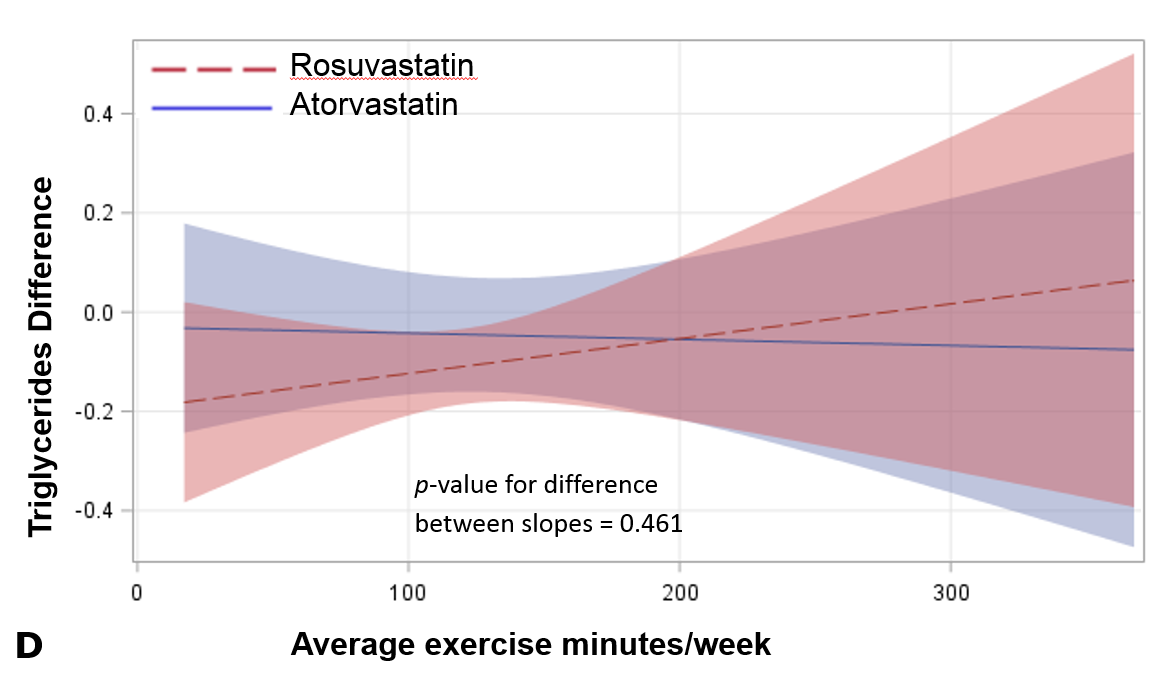


**Figure S2.** Adjusted model estimates and 95% CI of change in (A) Total cholesterol, (B) LDL, (C) HDL, and (D) triglycerides (in mmol/L) from baseline to the end of 12-week cardiac rehabilitation program by average exercise minutes per week and statin type used (using parametric test)
